# Estimation of Visual Maps with a Robot Network Equipped with Vision Sensors

**DOI:** 10.3390/s100505209

**Published:** 2010-05-25

**Authors:** Arturo Gil, Óscar Reinoso, Mónica Ballesta, Miguel Juliá, Luis Payá

**Affiliations:** Universidad Miguel Hernández, Avda. de la Universidad s/n, Ed. Quorum V, r03202 Elche (Alicante), Spain; E-Mails: o.reinoso@umh.es (O.R.); m.ballesta@umh.es (M.B.); mjulia@umh.es (M.J.); lpaya@umh.es (L.P.)

**Keywords:** visual SLAM, sensor fusion, uncertainty estimation, cooperative robots

## Abstract

In this paper we present an approach to the Simultaneous Localization and Mapping (SLAM) problem using a team of autonomous vehicles equipped with vision sensors. The SLAM problem considers the case in which a mobile robot is equipped with a particular sensor, moves along the environment, obtains measurements with its sensors and uses them to construct a model of the space where it evolves. In this paper we focus on the case where several robots, each equipped with its own sensor, are distributed in a network and view the space from different vantage points. In particular, each robot is equipped with a stereo camera that allow the robots to extract visual landmarks and obtain relative measurements to them. We propose an algorithm that uses the measurements obtained by the robots to build a single accurate map of the environment. The map is represented by the three-dimensional position of the visual landmarks. In addition, we consider that each landmark is accompanied by a visual descriptor that encodes its visual appearance. The solution is based on a Rao-Blackwellized particle filter that estimates the paths of the robots and the position of the visual landmarks. The validity of our proposal is demonstrated by means of experiments with a team of real robots in a office-like indoor environment.

## 1. Introduction

Mobile robots must possess a basic skill: the ability to plan and follow a path through the environment in an optimal way, while avoiding obstacles and computing its location within the map. In order to solve this problem, mobile robots require the existence of a precise map. In consequence, map building is an important task for autonomous mobile robots. This task is especially complex when no external measure of the robot location is available (e.g., no GPS signal is available). In such cases, the robot must face the situation in which it moves through an unknown space and incrementally builds a map of this environment, while simultaneously uses this map to compute its absolute location. In consequence, this problem has been designated as Simultaneous Localization and Mapping (SLAM) and has received great attention during the last decade. The SLAM problem is considered difficult, since an error in the estimation of the robot’s pose leads to an error in the map and *vice versa*. In this paper we consider the case in which the map building task is carried out simultaneously by a group of mobile robots that perform different trajectories in the environment. When multiple vehicles build the map simultaneously, this task will be finished more quickly and robustly than a single one [1], since the whole environment will be covered in less time. Also, at the same time more measurements can be obtained from the environment, thus giving the possibility to estimate a more precise map. However, in the multi-robot case, the SLAM problem becomes harder, since the trajectories of several robots need to be estimated and the dimensionality of the problem is increased.

SLAM techniques differ mainly in the kind of sensor used by the robot to obtain information from the environment. To date, typical SLAM approaches have used laser range sensors to build maps in two and three dimensions (e.g., [2–6]). Typically, these applications use directly the laser measurements to build 2D occupancy grid maps [2, 5], or they extract features from the laser measurements [4, 7] to build 2D landmark-based maps. Nevertheless, recently the interest on using cameras as sensors in SLAM has increased and researchers focus on the creation of three dimensional maps based on the measurements provided by vision sensors. These approaches are usually denoted as visual SLAM. Compared to laser ranging systems, stereo vision systems are typically less expensive. In addition, typical laser range systems allow to collect distance measurements on a 2D plane, whereas the information provided by stereo vision systems can be processed to provide a more complete 3D representation of the space. On the contrary, stereo systems are usually less precise than laser sensors. In common configurations, the camera is installed at a fixed height and orientation with respect to the robot reference system and the movement of the camera and robot is restricted to a plane [8, 9].

The research in visual SLAM has many similarities with the rich research in the Structure from Motion (SFM) field, carried out in the computer vision community. The approach taken in SFM, however, has generally been very different from visual SLAM solutions because the applications did not require real-time operation, and the trajectory of the camera and the structure of the environment could be computed offline. Some real-time SFM systems have been produced by efficient implementation of frame-to-frame SFM steps (e.g., [10]), in which repeatable localisation is possible and motion uncertainty does not grow without bound over time. Visual SLAM approaches typically deal with large camera trajectories and significant uncertainties in order to compute a visual map online.

Stereo vision systems provide a huge quantity of raw information from the environment stored in both images. In consequence, the images are normally processed in order to reduce the information to be used for mapping. As a result, most approaches to visual SLAM are feature-based. In this case, a set of points extracted from the images are used as visual landmarks. Features, such as image edges were used in [11] to build maps using a single camera. However, the localization of the camera with respect to the segments is difficult. For example, in [12] regions of interest are extracted using a visual attention system. The regions are extracted from images at different scales in a similar manner as the human perceptual system does. The main drawback with this kind of landmarks is that it may be difficult to obtain an accurate measurement of a region using a stereo camera, since regions can be arbitrarily large, thus providing inaccurate results.

In this paper we extract salient points from images and use them as visual landmarks in the environment. In a stereo system, corresponding points can be found in both images, thus obtaining a camera relative distance measurement. Mainly, two steps are distinguished in the selection of point-like visual landmarks. The first step involves the detection of interest points in the images that can be used as reliable landmarks. The points should be detected robustly at different distances and viewing angles, since they will be observed by the robot from different locations in the environment. At a second step the interest points are described by a feature vector, computed using local image information.

In the past, other authors have proposed different combinations of image detectors and descriptors in the context of mapping and localization. A summary of detection and description methods used in visual SLAM is included in Section 4. In order to compare the available methods, in a previous work [13] we evaluated the behavior of different interest point detectors and descriptors under the conditions needed to be used as landmarks in vision-based SLAM. To do this, we evaluated the repeatability of the detectors, as well as the invariance and distinctiveness of the descriptors under different perceptual conditions using sequences of images representing planar objects as well as 3D scenes. The results presented suggested that the Harris corner detector [14] in combination with the SURF (Speeded Up Robust Features [15]) descriptor outperformed other existing methods in terms of stability and discriminating power. In this sense, this paper can be understood as a prolongation of [13]. Thus, the real experiments presented here demonstrate the suitability of the selected detector and descriptor to compute 3D visual maps with a team of mobile robots in a real scenario.

In this paper we concentrate on the problem of cooperative visual SLAM and we propose a solution that allows to build a map using a set of visual observations provided by the sensors installed on every mobile robot. To date, most of the approaches to multi-robot SLAM are based on laser range sensors [2, 16]. However, in our opinion, little effort has been done until now in the field of multi-robot visual SLAM, which considers the case where several robots are equipped with vision sensors and are distributed in a robot network with the purpose of building a visual map. In addition, the suggested application requires the extraction of stable points from images in combination with a descriptor that uniquely describes each visual landmark. For example, consider the case in which two different robots use their sensors to observe the same visual landmark from two locations in the environment. In order to construct an accurate map both observations have to be associated with the same landmark in the map and this implies that the descriptor should be invariant to scale and general viewpoint transformations. In consequence, the difficulty of this problem suggested the evaluation of the existing point extraction methods and visual descriptors in order to find the most suitable to be used in visual SLAM [13].

As we said before, each observation obtained by the robot, has to be associated to one of the landmarks in the map. In this sense, the robot has to decide whether the observation corresponds to one of the landmarks previously integrated in the map, or, on the contrary, it is a new one. This situation is commonly referred as the data association problem, which is in close relation with the SLAM problem. We propose to use the visual description associated to the landmark along with the distance measurement obtained by the robot to solve the data association. Typically, when the robot moves along places already explored, it detects previously observed landmarks. In this case, if the current observations are correctly associated with the visual landmarks in the map, the robot will be able to find its location with respect to these landmarks, thus reducing the uncertainty in its pose. If the observations cannot be correctly associated to the landmarks in the map, the map will become inconsistent. Moreover, when several robots are exploring, the same landmark may be viewed simultaneously by the sensors of different robots from separate distances and angles. Still all the observations should be associated with the same landmark in the map. To sum up, the data association is a fundamental part of the SLAM process, since wrong data associations will produce incorrect maps and an inconsistent estimation of the paths.

Finally, it is worth noting that SLAM algorithms focus on the incremental construction of a map, given a set of movements carried out by the robots and the set of observations obtained from different locations, and they do not consider the computation of the movements that need to be performed by the robots. This is generally considered as a different problem, denoted as exploration. In this paper we concentrate on the visual SLAM problem and assume that the robots are able to explore the environment in an efficient way.

The main contribution of this paper consists in an approach that allows to solve the multi-robot SLAM problem using visual information by means of a Rao-Blackwellized particle filter (RBPF). In order to do this, we consider that each robot is equipped with a stereo camera sensor and is able to obtain 3D relative measurements to visual landmarks, each one accompanied by a descriptor. We also propose a method to solve the data association based on visual information that is well suited for the visual SLAM algorithm presented here.

This algorithm has been validated in indoor environments using real data obtained by a network of vision sensors installed on mobile robots. In addition, the SLAM algorithm has been tested under different conditions by changing the parameters in the filter. Evaluating the results of visual SLAM using real data is complex. The true path followed by the robots is not known, and the map cannot be known precisely, since the exact location of the landmarks cannot be established in advance. In consequence, we have compared the paths estimated by the visual SLAM algorithm with the paths estimated using laser range data. In this case, the ground truth is obtained using the laser-based approach described in [5], which has been shown to be very precise. The results demonstrate that our visual approach is suitable to build visual maps using small robot teams. In addition, the algorithm is suitable to be used in real time, that is, it can be used to build a map online while the robots explore the environment. We show results of two robots that explore the environment and simultaneously build a map of it.

The remainder of the paper is structured as follows. First, Section 2 presents related work in this field. Next, Section 3 presents the approach to multi-robot visual SLAM. Following, Section 4 introduces visual landmarks and deals with their utility in SLAM. Our approach to the data association problem in the context of visual features will be exposed in Section 5. Then, in Section 6 we present the experimental results obtained. Finally, Section 7 summarizes the most important conclusions.

## 2. Related Work

To date, the approaches to multi-robot SLAM can be classified in one of the two following groups:

- Distributed solutions: in this case each robot builds an independent local map using its own observations. Commonly, at a later step, the individual maps are referred to a global reference frame and fusion of the maps is carried out to build a common map.
- Centralized solutions: the estimation of the trajectories of the robots and the construction of the map are made by a centralized agent in the system. Normally, this agent receives the observations of all the robots in order to compute a single coherent map.

In [17] a distributed solution is presented. In that case, each robot builds its own local map and tries to find its location in the local maps built by other robots. The approach uses laser range finders and a particle filter localization method. The algorithm can cope with the situation where the initial locations of the robots are unknown. Nevertheless, the fusion of the individual maps is computationally expensive, since *K*^2^ particle filters must be maintained for a team of *K* robots.

A different approach is presented in [18]. In this solution, an extended Kalman filter (EKF) is used to jointly estimate the robot positions and a single map. In order to perform the estimation, the state vector is defined with the poses of all the robots in the team and the position of the landmarks. In this way, the natural extension of the classical EKF [19] to the multi-robot case allows to fuse the information of the sensors installed onboard of the vehicles and estimate a common map. In their approach, the relative location of the vehicles must be known in advance and the data association is assumed to be known. EKF-based solutions suffer from a main drawback: a single hypothesis over each robot pose is maintained. If false data associations are made the whole EKF may diverge [20]. Another example is presented in [21], using also an EKF to build a common map of features using the observations of every vehicle. The main difference with respect to [18] is that each robot builds a local map of the features in the vicinity and this local submap is then periodically fused into the global map. The main advantage of this representation is the reduction in computational time.

A different solution is exposed in [2]. It is based on the single-robot Rao-Blackwellized particle filter described in [5], but extending it to the joint estimation of the paths of several robots. In that paper two robots estimate simultaneously an occupancy grid map, considering the case in which the initial relative position of the robots is unknown. In [22] a multi-robot SLAM algorithm is presented that constructs 3D maps of point features. The SLAM algorithm is based on a Rao-Blackwellized particle filter extended to the joint estimation of every robot path and maps. The results simulate the navigation of a team of robots that extract visual landmarks from the environment and build a map of it. Simulation results are presented with teams of 2 to 4 members.

## 3. 3D Visual SLAM for a Team of Mobile Robots

In this section, the approach to multi-robot 3D visual SLAM is described. We consider the case in which a team of mobile robots explore the environment, and simultaneously build a map using visual information obtained from cameras.

The complexity of the SLAM problem stems from the fact that the estimation of the map robot poses are in close relation. As a result, if we make an error in the estimation of the pose, this induces an error in the estimation of the map and *viceversa*. On the other hand, the problem is made harder when more robots take part in the exploration, since the number of observations increases, as well as the dimensions of the state to estimate.

In the solution presented here we propose the use of a Rao-Blackwellized particle filter (RBPF), commonly referred as FastSLAM in the literature [4]. In our case, a Rao-Blackwellized particle filter represents the robot trajectories by means of a set of particles, combined with a closed estimation of the map conditioned to the trajectories.

We assume that every robot in the team has a stereo camera installed onboard, enabling them to compute the relative distance from the robot to visual landmarks found in the surroundings. In addition, each relative distance measurement is associated with a visual descriptor, computed using local information of the detected point in the images. In order to build the map, we also consider that the robots can exchange their information with a central agent in the system, sharing their observations performed over the landmarks to create a common map of the environment. We assume that, at the beginning of the exploration, the relative position of the robots is known.

Next, we explain the algorithm for the case where a single robot explores the environment and constructs a map with the 3D position of the observed landmarks. At time *t* the robot obtains an observation *o*_*t*_. The observation consists of two parts, *o*_*t*_ = (*z*_*t*_, *d*_*t*_), where *z*_*t*_ = (*X*_*c*_, *Y*_*c*_, *Z*_*c*_) is a three dimensional distance vector relative to the left camera reference frame and *d*_*t*_ is the visual descriptor associated to the landmark. The map *L* is represented by a collection of *N* landmarks *L* = {*l*_1_, *l*_2_, ..., *l*_*N*_ }. Each landmark is described as: *l*_*k*_ = {*μ*_*k*_, ∑_*k*_, *d*_*k*_}, where *μ*_*k*_ = (*X*_*k*_, *Y*_*k*_, *Z*_*k*_) is the mean vector that describes the 3D position of the landmark *k* in coordinates of a global reference frame. The mean vector is associated with the covariance matrix ∑_*k*_ that describes the uncertainty in the position of the landmark. Also, the landmark *l*_*k*_ is associated with a descriptor *d*_*k*_ that was computed using the visual appearance of the point in the space.

Next, we define the most usual nomenclature in Rao-Blackwellized SLAM. We denote the robot pose at time *t* as *x*_*t*_ and the movement of the robot at time *t* as *u*_*t*_. On the other hand, the robot path until *t* is referred as *x*_1:*t*_ = {*x*_1_, *x*_2_, . . . , *x*_*t*_}, the set of observations made by the robot until time *t* will be designated *z*_1:*t*_ = {*z*_1_, *z*_2_, . . . , *z*_*t*_} and the set of actions *u*_1:*t*_ = {*u*_1_, *u*_2_, . . . , *u*_*t*_}. The SLAM problem tries to determine the location of every landmark in the map *L* and robot path *x*_1:*t*_ using a set of sensor measurements *z*_1:*t*_ and considering that the robot performed the movements *u*_1:*t*_. Thus, the SLAM problem attempts to estimate the following probability function defined over the map and path:

(1) $$p\left(x_{1:t,}L|z_{1:t,}u_{1:t,}c_{1:t}\right)$$

where *c*_1:*t*_ designates the set of data associations performed until time *t*, *c*_1:*t*_ = {*c*_1_, *c*_2_, . . . , *c*_*t*_}. The data association is represented with the variable *c*_*t*_ and can be explained in the following manner: while the robot explores the map, it has to decide whether the observation *o*_*t*_ = (*z*_*t*_, *d*_*t*_) corresponds to a landmark observed before or it is a new one. Considering that, at time *t*, the map is formed by *N* landmarks, this correspondence is represented by *c*_*t*_, where *c*_*t*_ ∈ [1 *. . . N*]. The notation *z*_*t,c*_*t*__ means that the measurement *z*_*t*_ corresponds to the landmark *c*_*t*_ in the map. If none of the landmarks in the map until the moment correspond to the current measurement, we indicate it as *c*_*t*_ = *N* + 1, since a new landmark is added to the map. For the moment, we consider this correspondence as known. In Section 5 we present an algorithm to compute the data association using the data provided by the vision sensors.

We can find two different parts in the problem of SLAM: (i) the estimation of the trajectory of the robot. (ii) The estimation of the map. Both parts are closely related, since, the absolute position of a landmark in the map depends on the estimation of the location of the robot. Nevertheless, we can separate the two parts. For example, imagine that we could know the whole trajectory followed by the robot. In that case, it may be easy to build the map using the sensor measurements. We refer to this fact as the conditional independence property of SLAM problem. According to this property, the probability function defined in (1) can be written in the following manner [4]:

(2) $$p\left(x_{1:t,}L|z_{1:t,}u_{1:t,}c_{1:t}\right)=p\left(x_{1:t,}|z_{1:t,}u_{1:t,}c_{1:t}\right)\cdot \prod_{k=1}^{N}p\left(l_{k}|x_{1:t,}z_{1:t,}u_{1:t,}c_{1:t}\right)$$

that implies that the probability over the path *x*_1:*t*_ and the map *L*, given the observations *z*_1:*t*_, movements *u*_1:*t*_ and data associations *c*_1:*t*_ can be separated into two parts: the estimation of the probability over robot paths *p*(*x*_1:*t*_*|z*_1:*t*_, *u*_1:*t*_, *c*_1:*t*_), and the product of *N* independent estimators *p*(*l*_*k*_*|x*_1:*t*_, *z*_1:*t*_, *u*_1:*t*_, *c*_1:*t*_) over landmark positions, each conditioned to the path estimate . The first part *p*(*x*_1:*t*_*|z*_1:*t*_, *u*_1:*t*_, *c*_1:*t*_) is approximated using a set of *M* particles. Each particle stores *N* independent landmark estimations implemented as EKFs. Thus, we define each particle as:

(3) $$S_{t}^{\left[m\right]}=\left\{x_{1:t}^{\left[m\right]},\mu_{t,1}^{\left[m\right]},\Sigma_{t,1}^{\left[m\right]},d_{1}^{\left[m\right]},\ldots ,\mu_{t,N}^{\left[m\right]},\Sigma_{t,N}^{\left[m\right]},d_{N}^{\left[m\right]}\right\}$$

where $\mu_{t,k}^{\left[m\right]}$ represents the mean value for the position of landmark *l*_*k*_ conditioned to the path of the particle *m* and the observations until time *t*. On the other hand, $\Sigma_{t,k}^{\left[m\right]}$ refers to the covariance matrix associated to the uncertainty in the position. Finally, $d_{j}^{\left[m\right]}$ represents the visual descriptor associated to the landmark *j*. When a landmark is first sensed and initialized in the map a visual descriptor is computed in base of the image information nearby the projected point.

Whenever the robot moves, a new particle set $S_{t}=\left\{S_{t}^{\left[1\right]},S_{t}^{\left[2\right]},\ldots ,S_{t}^{\left[M\right]}\right\}$ is computed using the prior particle set *S*_*t*−1_ at time *t−*1 and the robot control *u*_*t*_. This, process represents the increase in uncertainty when the robot carries out a movement using the probability function *p*(*x*_*t*_*|x*_*t*−1_, *u*_*t*_). The estimation process follows a prediction and update fashion, as described in [22]. Each particle is assigned a weight that accounts for the probability of the estimated map and path.

Next, we present the algorithm that allows us to build a feature-based map while a team of *K* robots explores the environment equipped with vision sensors. We show the algorithm to estimate the map formed by the position of all the visual landmarks detected, as well as the trajectories followed by the robots during the exploration. In order to do so, we assume that, at time *t* the robot 〈*i*〉 is located at pose *x*_*t*,〈*i*〉_ and uses its vision sensor to obtain the observation *o*_*t*,〈*i*〉_ = {*z*_*t*,〈*i*〉_, *d*_*t*,〈*i*〉_}, where, again *z*_*t*,〈*i*〉_ is a relative measurement distance from the location of the robot to the observed landmark. Also, *d*_*t*,〈*i*〉_ denotes the visual descriptor associated with the landmark. The trajectory that the robot 〈*i*〉 followed until time *t* will be referred as *x*_1:*t*,〈*i*〉_ = {*x*_1,〈*i*〉_, *x*_2,〈*i*〉_, . . . , *x*_*t*,〈*i*〉_}.

Next, we need to represent the trajectories of all the robots in the team. In consequence, *x*_1:*t*,〈1:*K*〉_ = {*x*_1:*t*,〈1〉_, *x*_1:*t*,〈2〉_, . . . , *x*_1:*t*,〈*K*〉_} will represent all the trajectories of the robots until time *t*. In addition, *u*_1:*t*,〈1:*K*〉_ = {*u*_1:*t*,〈1〉_, *u*_1:*t*,〈2〉_, . . . , *u*_1:*t*,〈*K*〉_} denotes the movements performed by the robots and the observations will be referred as *z*_1:*t*,〈1:*K*〉_ = {*z*_1:*t*,〈1〉_, *z*_1:*t*,〈2〉_, . . . , *z*_1:*t*,〈*K*〉_}. In a similar way to the case where a single robot explores the environment, we express the data association with the variable *c*_*t*_, which indicates that the observation *z*_*t*,〈*i*〉_ was produced when the robot observed the landmark *c*_*t*_ of the map. It is important to highlight that we propose to estimate a single map common to all the robots, thus each observation will have a correspondence to a landmark in the map with independence of the robot that observed it. At each time step, the robot obtains an observation and computes the data association. We refer to the set of data associations until time *t* as *c*_1:*t*_ = {*c*_1_, *c*_2_, . . . , *c*_*t*_}.

Using the previous notation, we can define the multi-robot SLAM problem as that of estimating the probability density function:

(4) $$\begin{array}{lll} p\left(x_{1:t,\langle 1:K\rangle },L|z_{1:t,\langle 1:K\rangle },u_{1:t,\langle 1:K\rangle },c_{1:t}\right) & = & p\left(x_{1:t,\langle 1:K\rangle }|z_{1:t,\langle 1:K\rangle },u_{1:t,K\langle 1:K\rangle },c_{1:t}\right)\cdot \\ & & \prod_{k=1}^{N}p\left(l_{k}|x_{1:t,\langle 1:K\rangle ,}z_{1:t,\langle 1:K\rangle },u_{1:t,\langle 1:K\rangle },c_{1:t}\right) \end{array}$$

that poses the estimation of a set of *K* trajectories *x*_1:*t*,〈1:*K*〉_ and the map *L*, considering that the robots have performed a number of movements *u*_1:*t*,〈1:*K*〉_ and observations *z*_1:*t*,〈1:*K*〉_ with data association *c*_1:*t*_ in the map. Equation (4) is analogous to Equation (2) and indicates that the estimation of the map and the *K* trajectories can be separated into two parts: the first function *p*(*x*_1:*t*,〈1:*K*〉_*|z*_1:*t*,〈1:*K*〉_, *u*_1:*t*,〈1:*K*〉_, *c*_1:*t*_) suggests the estimation of the robot trajectories, conditioned to all the measurements, movements and data associations, whereas the map is estimated using *N* independent estimations conditioned to the paths *x*_1:*t*,〈1:*K*〉_. In consequence, Equation (4) divides the SLAM problem in two different parts: the localization of *K* robots in the map and a number of independent landmark estimations, each one conditioned to the trajectories *x*_1:*t*,〈1:*K*〉_ of all the robots. The estimation of both parts is carried out by means of a particle filter. Each of the *M* particles is associated with a map *L*, formed by *N* independent estimations for each of the landmarks. The computation of each landmark position is implemented by means of an Extended Kalman Filter (EKF). In our case, each of the Kalman Filters will be conditioned to the *K* paths of the robot team. Each particle in the filter is represented as:

(5) $$S_{t}^{\left[m\right]}=\left\{x_{1:t,\langle 1:K\rangle }^{\left[m\right]},\mu_{1,t}^{\left[m\right]},\Sigma_{1,t}^{\left[m\right]},d_{1}^{\left[m\right]},\cdots ,\mu_{N,t}^{\left[m\right]},\Sigma_{N,t}^{\left[m\right]},d_{N}^{\left[m\right]}\right\}$$

If we compare this definition with the particle defined in (3), we may observe that, in this case, the state to estimate is formed with the pose (x, y, *θ*) of each of the *K* robots in the team, being (x, y) the position of the robot in cartesian coordinates and *θ* the orientation of the robot. We denote the set of robot poses of the whole team as *x*_*t*,〈1:*K*〉_ = {*x*_*t*,〈1〉_, *x*_*t*,〈2〉_, ⋯,*x*_*t*,〈*K*〉_}. To sum up, the algorithm proposes the estimation of a path state of dimension 3*K* using jointly the information provided by all the sensors in the robot network. In order to clarify, Table 1 presents the structure of the particle defined in (5).

In the algorithm proposed here, a common map is shared by all the robots. This fact means that, when a robot changes the estimation of a landmark using its sensor measurement, this change affects the map of the whole robot team. The main advantage of this solution is that one member of the team may sense a landmark previously seen by a different robot and update its estimate, thus fusing the measurements obtained by both robot sensors. Also, another advantage is that a robot can reduce the uncertainty in its pose by observing landmarks previously mapped by other robots. As a consequence, the robots fuse all the information provided by its sensors in the estimation of the map and all the observations of every robot are used in the estimation of the map and paths.

In principle, in order to obtain a good estimation, the number of particles required increases exponentially with the dimension of the state [23]. Nevertheless, we present here results that show that the approach is suitable for robot teams of 2–3 members using a reasonable number of particles.

Next, we summarize the proposed algorithm. In order to clarify the ideas, the algorithm is separated in four different steps:

- New particles generation (Section 3.1).
- Updating each landmark with new sensor measurements (Section 3.2).
- Compute a weight for each particle (Section 3.3).
- Resampling based on the weight (Section 3.4).

We provide a detailed explanation of each step in the following subsections. In addition, in Algorithm 1 we describe the complete process, assuming that there exist three robots that explore simultaneously the environment.

### 3.1. New Particles Generation

In the beginning of the exploration, we assume that each robot knows its location with precision. Next, the robot moves and uses its odometry to estimate the new location in the map. Since the odometry is noisy, the robot is no longer certain of its position, thus the uncertainty over its pose has to be reproduced. In the proposed algorithm the particle set *S*_*t*_ represents the possible locations of the robot in the environment. When the robot moves, the uncertainty grows, and this fact is translated in a wider spread of the particles associated to each robot. In order to do this, we generate a new set of hypotheses *S*_*t*_ based on the previous set *S*_*t*−1_ by adding some noise according to a probabilistic motion model *p*(*x*_*t*_*|x*_*t*−1_, *u*_*t*_). We compute a new pose $x_{t,\langle i\rangle }^{\left[m\right]}$ for each one of the robots independently using the motion model, by applying the movement model to each of the *K* poses of the particle separately using *u*_*t*,〈*i*〉_, the movement performed by the robot 〈*i*〉.

### 3.2. Updating Each Landmark with New Sensor Measurements

We update the 3D position of each landmark using the pose of the robot 〈*i*〉 that used its sensor to obtain the observation *o*_*t*,〈*i*〉_ = {*z*_*t*,〈*i*〉_, *d*_*t*,〈*i*〉_}. For the moment we assume that this measurement belongs to the landmark *c*_*t*_. Later, in Section 5 we deal with this problem in more detail.

The computation of a new estimation for each landmark *l*_*k*_ is performed independently, using the standard EKF equations:

(6) $${\hat{z}}_{t,\langle i\rangle }=g\left(x_{t,\langle i\rangle }^{\left[m\right]},\mu_{c_{t,}t-1}^{\left[m\right]}\right)$$

(7) $$G_{l_{c_{t}}}=\nabla_{l_{c_{t}}}{g\left(x_{t,}l_{c_{t}}\right)}_{x_{t}=x_{t,\langle i\rangle }^{\left[m\right]};l_{c_{t}}=\mu_{c_{t,}t-1}^{\left[m\right]}}$$

(8) $$Z_{c_{t,}t}=G_{l_{c_{t}}}\Sigma_{c_{t,}t-1}^{\left[m\right]}G_{l_{c_{t}}}^{T}+R_{t}$$

(9) $$K_{t}=\Sigma_{c_{t,}t-1}^{\left[m\right]}G_{l_{c_{t},\langle i\rangle }}^{T}Z_{c_{t,}t}^{-1}$$

(10) $$\mu_{c_{t,}t}^{\left[m\right]}=\mu_{c_{t,}t-1}^{\left[m\right]}+K_{t}\left(z_{t,\langle i\rangle }-{\hat{z}}_{t,\langle i\rangle }\right)$$

(11) $$\Sigma_{c_{t,},t}^{\left[m\right]}=\left(I-K_{t}G_{l_{c_{t}}}\right)\Sigma_{c_{t,}t-1}^{\left[m\right]}$$

being *ẑ*_*t*,〈*i*〉_ the prediction for the current sensor measurement *z*_*t*,〈*i*〉_ that assumes that it has been associated with the landmark *c*_*t*_ in the map. The observation model *g*(*x*_*t*_, *l*_*c_t_*_) is linearly approximated by the Jacobian matrix *G*_*l*_*c*_*t*___, considering that the noise in the sensor is Gaussian and can be represented with the covariance matrix *R*_*t*_. Next, Equation (10) updates the prior estimate $\mu_{c_{t},t-1}^{\left[m\right]}$ of the landmark using the difference between the current and predicted observation *v* = (*z*_*t*,〈*i*〉_ *− ẑ*_*t*,〈*i*〉_). Following, Equation (11) represents the updated covariance matrix $\Sigma_{c_{t,}t}^{\left[m\right]}$, that defines the uncertainty in the landmark *c*_*t*_ associated to the particle *m*. In this case the covariance matrix is of dimensions 3 *×* 3, since $\mu_{c_{t},t-1}^{\left[m\right]}$ is a three dimensional vector representing the 3D position of the landmark *c*_*t*_.

Following, we define the matrix *R*_*t*_ associated with the noise in the sensor. In our case, we consider the equations of a standard stereo pair of cameras, assuming pin-hole cameras and parallel optical axes of both cameras. In a standard stereo pair of cameras, we can compute the 3D coordinates of a point in space relative to the left camera reference system as:

(12) $$X_{r}=\frac{I\left(c-C_{x}\right)}{d},Y_{r}=\frac{I\left(C_{y}-r\right)}{d},Z_{r}=\frac{fI}{d}$$

where, (*r*, *c*) are the coordinates in pixels (row and column) of the 3D point projected in the left image. The 3D point projects in the pixel (*r*, *c* + *d*) in the right image. The variable *d* denotes the disparity associated to that point. The parameter *I* is named baseline, and corresponds to the horizontal separation of both cameras and the parameters *C*_*x*_ and *C*_*y*_ refer to the intersection of the optical axis with the image plane in both cameras. Also, the parameter *f* is the focal distance of the cameras. Given that we assume an error in the estimation of the projected points (*r*, *c*), we can compute the propagation of this error to the relative measurement (*X*_*r*_, *Y*_*r*_, *Z*_*r*_), assuming a first order linear error propagation law. In consequence, the covariance matrix *R*_*t*_ associated to *z*_*t*,〈*i*〉_ = (*X*_*r*_, *Y*_*r*_, *Z*_*r*_) can be computed as: *R*_*t*_ = *JR*_*sp*_*J^T^*, where *J* = ▿_*r*,*c*,*d*_ *z*_*t*,〈*i*,*i*〉_ is the Jacobian matrix and *R*_*sp*_ is the error matrix associated with the errors in the computation of *d*, *r* and *c*. We assume typical errors in cameras that take into account the uncertainty in the camera calibration parameters: *σ*_*d*_ = 1 pixel, *σ_r_ = σ_c_* = 10 pixel. Thus $R_{\mathrm{sp}}=\mathrm{diag}\left(\sigma_{d}^{2},\sigma_{r}^{2},\sigma_{c}^{2}\right)$. This model has been previously used in a visual SLAM context in [24].

### 3.3. Compute a Weight for Each Particle

As described in [22], we assign a weight to each particle based on the quality of the matching between the current observation. Then, the sampling process generates a new set of particles *S*_*t*_ by choosing particles from the set *S*_*t*−1_. In the case of *K* robots, and considering that each one obtains a single measurement from its sensor *z*_*t*,〈*i*〉_ with data association *c*_*t*_, the weight $\omega_{t,\langle i\rangle }^{\left[m\right]}$ associated with the particle *m* and the robot 〈*i*〉 is computed as:

(13) $$\omega_{t,\langle i\rangle }^{\left[m\right]}=\frac{1}{\sqrt{\left|2\pi Z_{c_{t}}\right|}}e^{\left\{-\frac{1}{2}{\left(z_{t,\langle i\rangle }-{\hat{z}}_{t,c_{t}}\right)}^{T}{\left[Z_{c_{t}}\right]}^{-1}\left(z_{t,\langle i\rangle }-{\hat{z}}_{t,c_{t}}\right)\right\}}$$

Since, as defined in Equation (5), each particle $S_{t}^{\left[m\right]}$ represents the trajectories of *K* robots, we compute *K* weights *ω*_*t*,〈*i*〉_={*ω*_*t*,〈1〉_ ⋯ *ω*_*t*,〈*K*〉_}. In order to estimate the joint probability over the robot paths, the total weight associated to the particle $S_{t}^{\left[m\right]}$ is calculated by the product of the prior *K* weights: $\omega_{t}^{\left[m\right]}=\prod_{i=1}^{K}\omega_{t,\langle i\rangle }^{\left[m\right]}$. Finally, the weights are normalized to approximate a probability density function, so that $\Sigma_{i=1}^{M}\omega_{t}^{\left[i\right]}=1$.

### 3.4. Resampling Based on the Weight

During the resampling process, a particle from the set *S*_*t*−1_ will be included in the set *S*_*t*_ or not with probability proportional to its weight $\omega_{t}^{\left[m\right]}$. If the weight assigned to the particle is high, the particle will be included in *S*_*t*_ with high probability. Often, particles with low weight are discarded and are not integrated in *S*_*t*_. After resampling, the resulting particle set *S*_*t*_ is distributed according to *p*(*x*_1:*t*,〈1:*K*〉_, *L|z*_1:*t*,〈1:*K*〉_, *u*_1:*t*,〈1:*K*〉_, *c*_1:*t*,〈1:*K*〉_).

### 3.5. Path and Map Estimation

As described so far, the SLAM filter stores a set of particles that represent a set of hypothesis over the possible trajectories of every robot in the team. A number of 3D landmark positions is associated to each particle, being each landmark represented with an extended Kalman Filter. Since different trajectories and maps exist in the particle set, we need to choose the path and the map that best represent the true trajectories and map. In order to do this, we choose the most probable particle as the one that maximizes the sum:

(14) $$\hat{m}=\underset{m}{\mathrm{argmax}}\sum_{t=1}^{A}\text{log}\left(w_{t}^{\left[m\right]}\right)$$

being *A* the total number of steps performed by the robot along the path.

## 4. Visual Landmarks

In our case, our vision sensor provides the robot with a huge amount of information, generally encoded in the gray-level images. This quantity of information has to be reduced in order to accelerate the map building process. A common approach considers the use of visual landmarks. Thus, the construction of the map considers the estimation of the position of all the visual landmarks detected by the robots. A visual landmark can be defined as a point in space that can be easily detected using images obtained by the camera at different distances and viewing angles. We focus on natural visual landmarks, that is: any point that exists in the environment that can be extracted from images by means of a detection algorithm.

**Algorithm 1:** Summary of the algorithm.

1: *S* = ∅
2: [*Image*_*t*,〈1〉_, *Image*_*t*,〈2〉_, *Image*_*t*,〈3〉_] = AcquireImages ()
3: [*o*_*t*,〈1〉_, *o*_*t*,〈2〉_, *o*_*t*,〈3〉_] = ObtainObservations(*Image*_*t*,〈1〉_, *Image*_*t*,〈2〉_, *Image*_*t*,〈3〉_)
4: InitialiseMap(*S*, *x*_0,〈1:3〉_, *o*_*t*,〈1:3〉_)
5: **for***t* = 1 to numMovements **do**
6: [*Image*_*t*,〈1〉_, *Image*_*t*,〈2〉_, *Image*_*t*,〈3〉_] = AcquireImages ()
7: [*o*_*t*,〈1〉_, *o*_*t*,〈2〉_, *o*_*t*,〈3〉_] = ObtainObservations(*Image*_*t*,〈1〉_, *Image*_*t*,〈2〉_, *Image*_*t*,〈3〉_)
8: [*S*, *ω*_*t*,〈1〉_] = MRSLAM(*S*, *o*_*t*,〈1〉_, *R*_*t*_, *u*_*t*,〈1〉_)
9: [*S*, *ω*_*t*,〈2〉_] = MRSLAM(*S*, *o*_*t*,〈2〉_, *R*_*t*_, *u*_*t*,〈2〉_)
10: [*S*, *ω*_*t*,〈3〉_] = MRSLAM(*S*, *o*_*t*,〈3〉_, *R*_*t*_, *u*_*t*,〈3〉_)
11: *ω*_*t*_ = *ω*_*t*,〈1〉_*ω*_*t*,〈2〉_*ω*_*t*,〈3〉_
12: [*Neff*] = ComputeNeff(*ω*_*t*_)
13: **if***Neff <* 0.5 *//* resample when the number of effective particles is low? **then**
14: S = ImportanceResampling(*S*, *ω*_*t*_) *//* Sample randomly from *S*
15: **end if**
16: **end for**
** function** [*S*_*t*_] = **MRSLAM**(*S*_*t−*1_, *o*_*t*,〈*i*〉_, *R*_*t*_, *u*_*t*,〈*i*〉_)
17: *S*_*t*_ = ∅
18: **for***m* = 1 to *M* {repeat for every particle} **do**
19: $\left[x_{t,\langle i\rangle }^{\left[m\right]}\right]=\text{GenerateNewparticeWith Noise}\left(x_{t-1,\langle i\rangle },u_{t,\langle i\rangle }\right)$*//* generate a new particle using the movement model *p*(*x*_*t*,〈*i*〉_*|x*_*t−*1,〈*i*〉_, *u*_*t*,〈*i*〉_)
20: **for**$n=1\;\text{to }N_{t-1}^{\left[m\right]}$ // find data associations **do**
21: ${\hat{z}}_{t,\langle i\rangle }=g\left(x_{t,\langle i\rangle }^{\left[m\right]},\mu_{n,t-1}^{\left[m\right]}\right)$
22: $G_{l_{n}}=\nabla_{l_{c_{t}}}g{\left(x_{t},l_{n}\right)}_{x_{t,\langle i\rangle }=x_{t,\langle i\rangle }^{\left[m\right]};l_{c_{t}}=\mu_{c_{t,}t-1}^{\left[m\right]}}$
23: $Z_{n,t}=G_{l_{n}}\Sigma_{n,t-1}^{\left[m\right]}G_{l_{n}}^{T}+R_{t}$
24: *D*(*n*) = (*z*_*t*,〈*i*〉_*− ẑ*_*t*,〈*i*〉_)*^T^* [*Z*_*n*,*t*_]*^−^*^1^(*z*_*t*,〈*i*〉_*− ẑ*_*t*,〈*i*〉_)
25: *E*(*n*) = (*d*_*t*,〈*i*〉_*− d*_*n*_)*^T^* (*d*_*t*,〈*i*〉_*− d*_*n*_)
26: **end for**
27: $D\left(N_{t-1}^{\left[m\right]}+1\right)=D_{0}$
28: *j* = *find*(*D* ≤ *D*_0_) {Find candidates below *D*_0_}
29: *c*_*t*_ = *argmin*_*j*_*E*(*j*) {Find minimum among candidates}
30: **if***E*(*c*_*t*_) > *E*_0_*//* Is this a new landmark? **then**
31: $c_{t}=N_{t-1}^{\left[m\right]}+1$
32: **end if**
33: **if**$c_{t}=N_{t-1}^{\left[m\right]}+1$ / / New landmark **then**
34: $N_{t}^{\left[m\right]}=N_{t-1}^{\left[m\right]}+1$
35: $\mu_{c_{t},t}^{\left[m\right]}=g^{-1}\left(x_{t,\langle i\rangle }^{\left[m\right]},z_{t,\langle i\rangle }\right)$
36: $\Sigma_{c_{t},t}^{\left[m\right]}=G_{l_{c_{t}}}^{T}R_{t}^{-1}G_{l_{c_{t}}}$
37: $\omega_{t}^{\left[m\right]}=p_{0}$
38: **else**
39: $N_{t}^{\left[m\right]}=N_{t-1}^{\left[m\right]}//\;\text{Old landmark}$
40: $K_{t}=\Sigma_{c_{t},t-1}^{\left[m\right]}G_{l_{c_{t}}}^{T}Z_{c_{t},t}^{-1}$
41: $\mu_{c_{t},t}^{\left[m\right]}=\mu_{c_{t},t-1}^{\left[m\right]}+K_{t}\left(z_{t,\langle i\rangle }-{\hat{z}}_{t,\langle i\rangle }\right)$
42: $\Sigma_{c_{t},t}^{\left[m\right]}=\left(I-K_{t}G_{l_{c_{t}}}\right)\Sigma_{c_{t},t-1}^{\left[m\right]}$
43: **end if**
44: $\omega_{t,\langle i\rangle }^{\left[m\right]}=\frac{1}{\sqrt{\left|2\pi Z_{c_{t}}\right|}}e^{\left\{-\frac{1}{2}{\left(z_{t,\langle i\rangle }-{\hat{z}}_{t,c_{t}}\right)}^{T}{\left[Z_{c_{t}}\right]}^{-1}\left(z_{t,\langle i\rangle }-{\hat{z}}_{t,c_{t}}\right)\right\}}$
45: add $\left\{x_{t,\langle i\rangle }^{\left[m\right]},N_{t}^{\left[m\right]},\mu_{1,t}^{\left[m\right]},\Sigma_{1,t}^{\left[m\right]},d_{1,t}^{\left[m\right]},\cdots ,\mu_{N_{t}^{\left[m\right]},t}^{\left[m\right]},\Sigma_{N_{t}^{\left[m\right]},t}^{\left[m\right]},d_{N_{t}^{\left[m\right]},t}^{\left[m\right]},\omega_{t}^{\left[m\right]}\right\}$ to *S*_*t*_
46: **end for**
47: **return***S*_*t*_

Two main processes can be found in the observation of a visual landmark: the detection phase and the description phase. The detection considers the extraction of a set of points from images of the environment. In the case of visual landmarks, we aim at detecting some points in the images that are highly salient, and can be easily detected at different distances and viewing angles. The description of the landmark aims at representing the landmark based on its visual appearance. The descriptor enables the robot to recognize a particular landmark in the map, thus, it is in close relation with the data association problem. The description of the visual landmarks is based on a sub-image centered at the detected point.

In the context of mapping and localization different combinations of detectors and descriptors have been used. For example, in the context of monocular SLAM, in [25] the Harris corner detector was used to find salient points in images and describe them using a window patch centered at the detected points. In [26] the SIFT transform was presented, which combines an interest point detector and a description method, initially applied to object recognition applications [27]. Later, in [24] SIFT features were used as visual landmarks in the 3D space in a map building applications. In [8] the detected SIFT points were tracked to keep the most robust ones. Other authors [28] used a rotationally variant version of SIFT in combination with a Harris-Laplace detector for monocular SLAM. The SIFT transform has been used widely, for example [29] presents the applications of the SIFT transform to photogrammetric applications, where the extracted points are used to match aerial images.

Recently, the SURF features were presented [15], which also proposes an interest point detector in combination with a descriptor. Lately, in [30] the SURF features were used in localization tasks using omnidirectional images.

In the context of matching and recognition, many authors have presented their works evaluating several interest point detectors and descriptors. For example, in [31] a collection of detectors is evaluated by measuring the quality of these features for tasks like image matching, object recognition and 3D reconstruction. However, the mentioned work does not consider the interest point detectors that are most frequently used nowadays in visual SLAM.

Several comparative studies of local region detectors and descriptors have been presented so far. For instance, [32] present a comparison of several local affine region detectors. On the other hand, in [33] different detectors are used to extract affine invariant regions, but they focus on the comparison of different description methods. A set of local descriptors are evaluated using a criterion based on the number of correct and false matches between pairs of images. All these previous approaches perform the evaluation of detection and description methods using pairs of images and analyze different imaging conditions.

The case of visual SLAM is particularly difficult, since some point extractors fail at detecting the same point when viewed from different distances or angles. In consequence, it is difficult to detect again previously mapped landmarks, for example, when the robot observes the scene from a different pose. Moreover, the description of the points must be invariant to changes in viewing distance (scale) and viewing angle. In the case of visual landmarks it is difficult to compute a visual description that is totally invariant, since the appearance of a point in space varies largely with viewpoint changes. In consequence, the data association problem becomes hard to solve. For this reason, in a prior work [13], a comparison of point detectors and descriptors was carried out. The study focuses on the comparison of different interest points detectors and descriptors under the conditions needed to be used as landmarks in vision-based SLAM. The repeatability of different interest point detectors, as well as the invariance and distinctiveness of several description methods is evaluated. The evaluation is made in conditions similar to the case of visual SLAM. In order to do this, sequences of images representing planar objects as well as 3D scenes were used. It is worth noting that in this study the Harris corner detector obtained the best results for the kind of indoor images used. Moreover, the U-SURF descriptor showed good properties to be used as a descriptor for visual SLAM. The U-SURF descriptor is a particular version of the SURF descriptor. In consequence, this fact justifies the combination of descriptor and detector used in the experiments presented here.

## 5. Data Association

**Figure 1.**
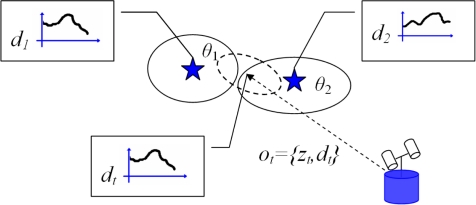
The figure describes the data association problem in the context of visual landmarks.

When the robot moves along the environment, it captures images with its sensor and processes them to obtain observations. As described previously, the SLAM algorithm needs to decide whether the observation *o*_*t*,〈*i*〉_ = {*z*_*t*,〈*i*〉_, *d*_*t*,〈*i*〉_} was obtained from an already seen landmark in the map or it is a new landmark that should be initialised. In this section, we introduce a method to compute the data association and the extension to the multi-robot case. Figure 1 explains the data association in the case of visual landmarks. In the figure, we represent the position of two different visual landmarks with stars, with uncertainty depicted with an ellipse. Each landmark (*θ*_1_, *θ*_2_) owns a visual descriptor (*d*_1_, *d*_2_). With dashed line we represent the observation *o*_*t*,〈*i*〉_ = {*z*_*t*,〈*i*〉_, *d*_*t*,〈*i*〉_} that consists of a distance measurement *z*_*t*,〈*i*〉_ and a visual descriptor *d*_*t*,〈*i*〉_. The uncertainty in the measurement is denoted by an ellipse. Since we are using a vision sensor, the descriptor *d*_*t*,〈*i*〉_ describes the visual appearance of the point in space. In this case, the robot must decide whether the observation *o*_*t*,〈*i*〉_ corresponds to landmark *θ*_1_, landmark *θ*_2_ or it is a new landmark. In [4] a Mahalanobis distance function is used in the computation of the data association. Thus, the distance *D* is computed for all the landmarks in the map:

(15) $$D={\left(z_{t,\langle i\rangle }-{\hat{z}}_{t,c_{t}}\right)}^{T}{\left[Z_{c_{t}}\right]}^{-1}\left(z_{t,\langle i\rangle }-{\hat{z}}_{t,c_{t}}\right)$$

In the this approach, the measurement *z*_*t*,〈*i*〉_ is associated with the landmark *c*_*t*_ in the map that minimizes *D*. If the minimum value surpasses a pre-defined threshold, a new landmark is created.

According to [20] the previous solution fails if the landmarks are placed close to each other in the environment, which occurs frequently in the case of visual SLAM, leading to a high number of false data associations. In consequence, we propose to improve this scheme by incorporating the visual description of the landmark in the data association process. In our case, we will employ a U-SURF descriptor associated to each landmark in the map. The data association is computed in the following way. First, we compute the distance *D* for all the landmarks in the map and select those that are below a threshold *D*_0_ as candidates. Next, given a U-SURF descriptor as observed by the robot *d*_*t*,〈*i*〉_ and a U-SURF descriptor *d*_*j*_ associated to a landmark in the map, we compute a squared Euclidean distance function:

(16) $$E=\left(d_{t,\langle i\rangle }-d_{j}\right){\left(d_{t,\langle i\rangle }-d_{j}\right)}^{T}$$

The distance *E* is computed for the list of candidates. Next, we select the landmark in the map that minimizes *E*. If the distance *E* is below a threshold *E*_0_ the observation is associated with the landmark. On the other hand, a new landmark is created whenever the distance *E* exceeds a pre-defined threshold (selected experimentally), since the current observation cannot be correctly explained by any of the landmarks in the map up to now.

The data association is performed independently for each particle, and this means that different particles would make different data associations. In addition, that means that the association method must be fast, since it needs to be computed for each particle. In a previous work [8], we used this approach in the case of visual SLAM with a single robot.

## 6. Results

**Figure 2.**
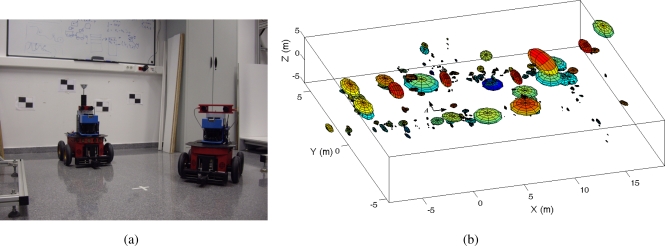
Figure (a): robots used in the experiments; Figure (b): visual map created jointly using two robots.

In this section we present results using real data acquired using Pioneer P3-AT robots in an indoor environment. Figure 2(a) shows the robot equipped with the SICK laser range finder and the stereo head onboard. The STH-MDCS2-VARX (Videre Design) stereo head is placed at a fixed angle on the robot. The experiments were performed at Miguel Hernández University in a typical office indoor environment. Mainly, we present two different experiments: first, we present results obtained by commanding the robot along a given trajectory in the environment (Section 6.1). Second, we also show results of our visual SLAM algorithm working in an online exploration application (Section 6.2).

### 6.1. Results Offline

In the first case, we commanded a single robot along the environment following different trajectories. Each trajectory begins from a different starting point. Two 640 *×* 480 stereo images are captured whenever the robot moved a given distance or rotated a given angle. Next, Harris points are extracted at each image and, for each point an U-SURF descriptor is computed. In addition, the stereo correspondence is found in the stereo images, obtaining a three dimensional distance vector for each point. Moreover, the measurements are tracked for a number of frames in order to extract robust points. The points that can be found in a number of successive robot poses are integrated in the SLAM filter.

In order to build the visual map, we use trajectories captured at different times by the same robot, emulating the case were different robots explore the environment simultaneously. The experiments include significant changes in illumination, due to natural solar changes. Thus, this further increases the difficulty of the problem. Nevertheless, the results show that the robots are capable of detecting and associating the same landmarks in the environment and build a correct map. In addition, laser range data and odometry readings were stored. These data were processed using the algorithm described in [5] in order to build an occupancy grid map and compute the robot path. Since the laser-based algorithm has demonstrated to be very precise, we decided to use it as ground truth data.

The origin of the trajectory of each robot is known in advance when the robots start exploring the environment. One of the robots is considered to start at the origin of the reference system common to all the robots. The initial pose of the rest of the vehicles is referred to this reference system. Since the initial position of the rest of the team is known approximately, the particles are initialized using a gaussian distribution around the starting poses.

Next, we present results of mapping when using the trajectories of two robots simultaneously. For example, Figure 2(b) shows a visual map built using the observations of two robots simultaneously and the multi-robot visual SLAM proposed. The position of each visual landmark is indicated with an ellipse with size proportional to the uncertainty in its location. In the maps, we indicated the origin of the common reference system as “A”. The relative starting position of the other robot respect to this reference is (x, y, *θ*) = (*−*4, 0, 0) *m*. In this case *M* = 1500 particles were used and *B* = 20 observations were integrated at each iteration of the algorithm.

**Figure 3.**
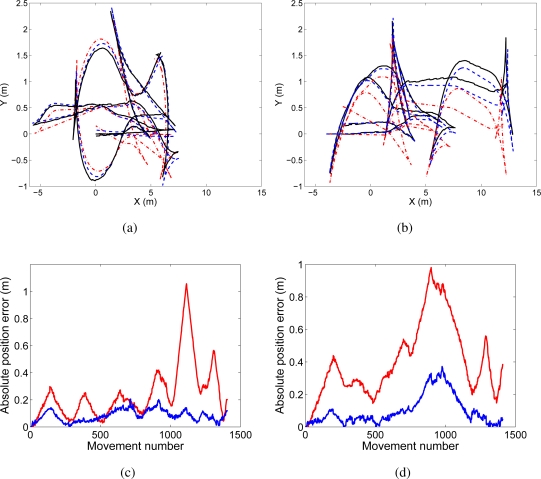
Figure (a) shows the true path (continuous line), odometry (dash and dots) and estimated path (discontinuous line) for the trajectory “A”. Figure (b) presents the true path (continuous line), odometry (dash and dots) and estimated path (discontinuous line) for the trajectory “B”. Figure (c) presents the absolute position error in trajectory “A” at each time step, whereas Figure (d) presents the absolute position error in trajectory “B” at each time step.

The trajectories followed by the robots are presented in Figure 3. Figure 3(a) shows the path of the robot that will be denoted as trajectory “A” . The true path is shown in continuous line, the estimated path using visual SLAM is shown with dashed line, and the odometry is represented with dash-dotted line. In Figure 3(b) we present the same information for a different robot, that will be referred as trajectory “B”. In Figure 3(c) and 3(d) the absolute error in position is presented for both trajectories “A” and “B” respectively. In red color we show the error in odometry, whereas in blue we present the error in the estimated path.

**Figure 4.**
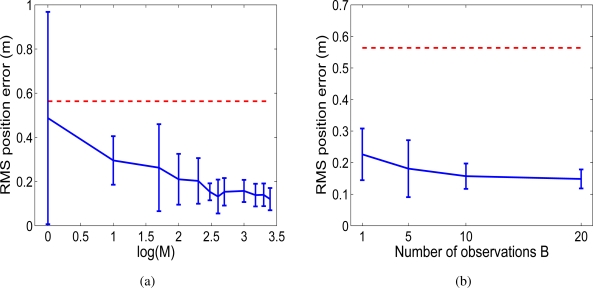
Figure (a): RMS error in position when the number *M* of particles is varied. Using *M* = {1, 10, 50, 100, 200, 300, 400, 500, 1000, 1500, 2000, 2500}. Figure (b): RMS error in position when the number *B* of observations integrated at each time step is varied *B* = {1, 5, 10, 20}.

Additionally, we changed the parameters in the Rao-Blackwellized particle filter and analysed the results. In Figure 4 we show the RMS position error in the trajectories when the number of particles *M* is changed. We can observe that the error decreases as the number of particles increases, since the probability distribution is represented more precisely with a higher number of particles. Nevertheless, a good estimation can be obtained using a reasonable number of particles. On the other hand, Figure 4(b) shows the RMS position error when the number of observations *B* is changed. It can be appreciated that the error decreases as the number of measurements integrated at each time step increases. In this way, the effect of the error introduced by one observation is reduced.

**Figure 5.**
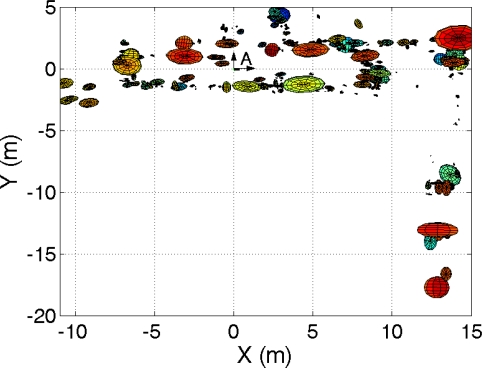
2D view of the visual map built jointly with three robots, as seen from above.

**Figure 6.**
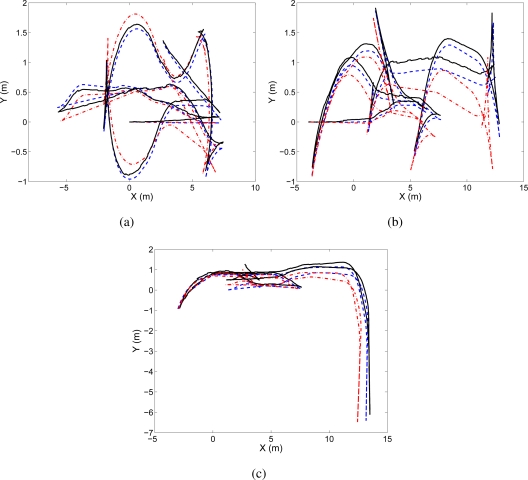
Figure (a): true path (continuous line), odometry (dash-dotted line) and estimated path (discontinuous) for trajectory “A”. Figure (b) and Figure (c) present the same data for trajectories “B” and “C” respectively.

In the following we present the results obtained using a team of three robots. We consider that the robots follow trajectories “A”, “B” and “C”. Also, in this case, the true path of the robots was obtained using laser range data. For example, in Figure 5 a map built using the observations of three robots is presented. We used *M* = 3000 particles and *B* = 20 observations at each time step. Figure 5 presents a 2D view of the visual map. On the other hand, in Figure 6 the true path, odometry and estimated path are presented.

In order to evaluate the algorithm in different conditions we used different trajectories with different starting positions. Next, we used the trajectories to build maps using the presented approach. We have observed that the algorithm is capable of building visual maps in real situations.

### 6.2. Results Online

Next, we present a set of results obtained in an exploration application. In this case, the robots were commanded by the common computer to explore the environment, using the exploration algorithm presented in [34]. While the robots move, they capture 640 *×* 480 stereo images and process them onboard, extracting Harris points and computing U-SURF descriptors. In addition, the visual landmarks are tracked locally. Only the observations found in 3 consecutive robot poses are integrated in the filter. The robots communicate to a common computer, where the proposed SLAM algorithm runs. The observations obtained by the robots are sent to this central system that is in charge of processing the observations to obtain a visual map and estimate the robot paths. Based on the position of each robot, the system computes the exploration commands needed to direct the robots to explore new places or return to previously explored places. In the experiments we used *M* = 500 particles and limited the number of observations that are integrated at each iteration of the filter to *B* = 8. This restrictions are necessary since the objective is to command the robots in real time while, at the same time, building the map. The parameters were selected experimentally. The robots move with a maximum translational speed of 0.05 *m/s* and rotate with a maximum speed of 0.03 *rad/s*. Each robot obtains observations whenever it translates more than 0.1 *m* or rotates more than 0.03 *rad*. The exploration algorithm requires the pose of all the robots in the team at all times. In consequence, this fact restricts the time needed to integrate the measurements in the visual SLAM algorithm.

To maintain the synchronization between the actual path of the robots and the path estimated, the visual SLAM algorithm must be capable of processing the observations of all the robots with a maximum delay of 0.1*/*0.05 = 2 *s*. Whenever the synchronization between the actual robot pose and the estimated path is lost, the robots stop their movements. We will show in the results that, with an adequate set of parameters in the visual SLAM filter, this situation occurs rarely.

**Figure 7.**
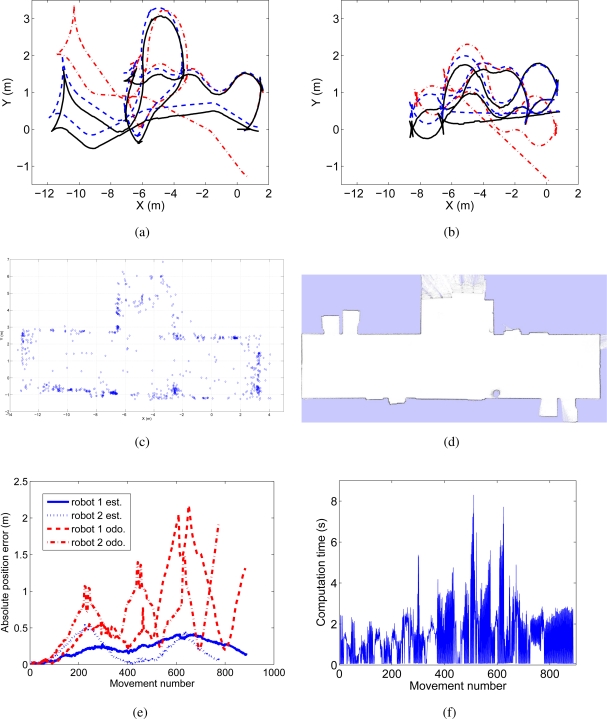
Results obtained in an online multi-robot visual SLAM experiment.

In Figure 7(a) and 7(b) the trajectories performed by both robots are presented. We present with continuous line the estimated path using laser range data, whereas the path estimated with the proposed approach is shown with discontinuous line. The trajectories given by the odometers are presented with dashes and dots. Next, in Figure 7(c) we present a detail of the visual map created. This map can be compared with an occupation grid built using laser data in Figure 7(d), since some of the visual landmarks are placed on the walls in the environment. Figure 7(e) presents the absolute position error at every step of the algorithm and is compared with the error in the odometry. The time needed by the visual SLAM algorithm at each iteration is presented in Figure 7(f). At each iteration, the algorithm integrates the observations of 2 robots. In this case, the mean execution time is 1.2 *s*. It can be observed that, in some cases, the execution time is over 2 *s*, which where fixed to maintain the synchronization between the actual path of the robots and the estimated path. During the experiments, we observed that this situation was not frequent and the robots managed to explore a 17 *×* 8 *m* environment in about 12 *min*.

## 7. Conclusions

We have presented an approach to visual SLAM that builds a 3D visual map of the environment using a team of cooperative robots. We consider that the robots are equipped with stereo sensors that allow to obtain observations over visual landmarks. These natural landmarks are extracted from images of the environment using the Harris corner detector. Next, each point is described using the U-SURF descriptor. Thus, each observation consists of a relative 3D distance measurement and a visual descriptor. In addition, the observations are tracked for a number of frames in order to obtain the most robust landmarks. The election of the detector and descriptor is based on a prior work presented in [13]. In this work, the detectors and descriptors were evaluated using sequences of images, emulating a visual SLAM scenario. In this case the Harris corner detector in combination with the U-SURF descriptor outperformed other combinations. In this paper, we have showed that the combination is suitable to build visual maps precisely.

The proposed visual SLAM approach builds a unique map of the environment that is shared by all the robots in the team, using a Rao-Blackwellized particle filter to estimate both the map and the trajectories of the robots. The uncertainty of all the robot team is maintained using a particle representation that encodes *M* hypotheses over the paths. In consequence, each particle represents a hypothesis over the paths of the robot team. In addition, there is a map associated to each particle conditioned to the paths and observations of all the vehicles. We consider that, initially the relative positions of the robots are known approximately. However, it is not necessary that the robots are placed in nearby poses at the start of the exploration.

The data association problem is solved using the relative measurements obtained by the robots and also comparing the visual descriptor. With this strategy we have found that the number of false correspondences is low, thus allowing to obtain good estimations of the map and the path. The data association is made for each particle independently of the rest, thus this fact requires a data association mechanism that can be computed rapidly. Since the robots share a common visual map, a robot may observe landmarks that were introduced in the map by a different robot to localize itself with respect to them.

We have tested the validity of the approach using real data captured by standard mobile robots equipped with a stereo head. The paths estimated using the proposed approach have been compared with the paths estimated using a laser-based SLAM approach. The reason for this choice is that the laser-based approach has demonstrated to be precise under the conditions of the experiments carried out in this work.

We have presented two different kind of results. First, data were gathered using a single robot that was commanded in the environment. Next, maps were built using different trajectories taken at different times, emulating the trajectories of different robots moving simultaneously. The results demonstrate that visual maps can be built with precision in different situations. The results presented show that the algorithm is robust to false data associations, and is able to produce a good solution with a wide range of parameter settings. Second, we present results of the multi-robot visual SLAM algorithm running online. In this case, the observations obtained by the robots are integrated in the visual SLAM filter simultaneously while the robots move along the environment. The estimated poses are used by a exploration algorithm to conduct the robots efficiently along the environment.

As a conclusion, under the assumptions considered, we have demonstrated that the proposed visual SLAM algorithm is suitable for small groups of robots exploring a given environment and obtaining observations over visual landmarks. We show that precise results can be obtained, maintaining a reasonable computational cost.

## Figures and Tables

**Table 1.** Particle set *S*_*t*_. Each particle is accompanied by *N* Kalman Filters.

Particle 1	{(*x*, *y*, *θ*)_〈1〉_, ⋯,(*x*, *y*, *θ*)_〈*K*〉_}^[1]^	$\mu_{1}^{\left[1\right]}\Sigma_{1}^{\left[1\right]}d_{1}^{\left[1\right]}$	⋯	$\mu_{N}^{\left[1\right]}\Sigma_{N}^{\left[1\right]}d_{N}^{\left[1\right]}$
⋮				
Particle *M*	{(*x*, *y*, *θ*)_〈1〉_, ⋯, (*x*, *y*, *θ*)_〈*K*〉_}^[^*^M^*^]^	$\mu_{1}^{\left[M\right]}\Sigma_{1}^{\left[M\right]}d_{1}^{\left[M\right]}$	⋯	$\mu_{N}^{\left[M\right]}\Sigma_{N}^{\left[M\right]}d_{N}^{\left[M\right]}$
